# Association of TCF7L2 Gene Polymorphisms with T2DM in the Population of Hyderabad, India

**DOI:** 10.1371/journal.pone.0060212

**Published:** 2013-04-05

**Authors:** Kommoju Uma Jyothi, Maruda Jayaraj, Kadarkarai Samy Subburaj, Kotla Jaya Prasad, Irgam Kumuda, Velaga Lakshmi, Battini Mohan Reddy

**Affiliations:** 1 Molecular Anthropology Group, Biological Anthropology Unit, Indian Statistical Institute, Hyderabad, India; 2 JP Endocrine Center, Ashok Nagar, Hyderabad, India; 3 Department of Human Genetics, Andhra University, Visakhapatnam, India; Central China Normal University, China

## Abstract

We attempt to evaluate the nature of association of TCF7L2 gene variants with T2DM, for the first time in the population of Hyderabad, which is considered to be diabetic capital of India. It is a case-control study of the three SNPs of TCF7L2, rs7903146, rs12255372 and rs11196205, genotyped on Sequenom Massarray platform, in a sample of 758 patients and 621 controls. The risk allele frequency of the three SNPs was found to be significantly higher in the T2DM cases than controls, implicating susceptibility for diabetes (p<0.01). The greatest risk of developing the disease was conferred by rs7903146. Further, the logistic regression of genotypes of each SNP under log additive model, and the haplotypes constituted by at least one of the three risk alleles also show significantly greater risk of developing T2DM when compared to the wild type haplotype. Further, BMI and WHR emerge as significant covariates with confounding effects. The strong association of the TCF7L2 SNPs with T2DM is consistent with the findings among other Indian and Non-Indian populations, suggesting universal phenomena of its association across ethnic groups globally, both within and outside the Indian subcontinent, albeit the functional relevance of these SNPs needs yet to be established.

## Introduction

Type 2 diabetes mellitus (T2DM) is the most common form of diabetes characterized by hyperglycemia, which is caused by impairment in both insulin secretion and action. It is a chronic disease leading to various complications such as coronary heart disease, diabetic nephropathy, neuropathy and retinopathy. It is becoming an epidemic with increasing prevalence throughout the world. Both genetic and environmental factors play a strong role in the manifestation of this complex genetic disorder. Unique in genetic pre-disposition of its population to diabetes, coupled with rapid urbanization, India is one of the leading countries presenting with largest number of diabetics (62.4 million). This number is increasing at an alarming rate and expected to touch 100 million by the year 2030 [1], [2]. The development of high throughput micro-array platforms unraveled a large number of genes involved in the etiology of T2DM. Among them, TCF7L2 *(Transcription factor 7 like 2)* is considered as one of the most important candidate genes which plays a major role in blood-glucose homeostasis and beta cell function. Strong association of TCF7L2 with T2DM was initially found in Icelandic population which has been subsequently replicated in Danish and U.S populations [3]. The three TCF7L2 SNPs (rs7390146, rs12255372 and rs11196205) that were strongly associated with T2DM in the above study were subsequently replicated, along with other SNPs of TCF7L2, in a huge meta-analysis [4] prompting their inclusion in any future replication effort.

The first GWAS study on T2DM in the French population showed strong signal for TCF7L2 [5]. Its association, along with the other T2DM genes, was also confirmed in subsequent GWAS studies. The consistency in the findings of its association with T2DM observed among many studies of diverse ethnic groups was considered indicative of a universal contribution of this gene to T2DM [6], albeit a few studies showed weak or no association with T2DM [7]–[12], which can be attributed to the extremely low frequency of the risk alleles and inadequate sample size, hence lack of power of the study.

The replicative studies of this candidate gene among a couple of Indian populations also showed strong association of TCF7L2 with T2DM but given enormous ethnic, cultural, geographic and genetic heterogeneity, large population size and relatively high prevalence of diabetes, very small and insignificant number/proportion of Indian populations were hitherto studied [13]–[17]. Further, these studies were confined to select regions like Pune, Punjab, Haryana, Himachal Pradesh, Delhi, Jammu & Kashmir and Chennai. Most of these populations, except that of Chennai come from northern parts of India and belong to the Indo-European linguistic family and considered to be quite distinct from those of Southern India- ethnically, linguistically and genetically [18]. Therefore, there is an urgent need to screen populations of the other Indian regions in order to project a representative scenario for the Indian subcontinent as a whole. It is also necessary to study the effect of environment and ethnicity on the T2DM susceptible genes to better appreciate the ‘Asian Indian phenotype’ that is uniquely predisposed to develop the disease. The Hyderabad city, in particular, which is considered as diabetic capital of India with high prevalence of T2DM, has not been explored and to the best of our knowledge, this is the first genetic study on T2DM in the population of Hyderabad or Andhra Pradesh. We present here the results of our analysis of the three SNPs of TCF7L2 (rs7903146 - intron 3, rs12255372 and rs11196205 - intron 4) for their association with T2DM.

These three SNPs, individually and/or as a group, were found to be associated with T2DM in a wide spectrum of world populations: Finnish [19], Amish [20], U.S [21], Polish [22], Scandinavian [23], Chinese [24], Brazil [25], Japanese[26]–[28], Swedish [29], U.K [30], Dutch [31], Palestinian [32], Iranian [33], [34], Arab [35], Tunisian [36], Persian [37] as well as Indian populations [13]–[17]. Of the three SNPs, rs7903146 is the most consistent in several GWAS studies [5], [38]–[45] as well as most replicated variant of TCF7L2. We present here the results of our analyses of association of the above three SNPs of TCF7L2 gene with T2DM in a large sample of patients and controls from the population of Hyderabad, which is considered as the diabetic capital of India.

## Materials and Methods

This study is based on a total sample of 1379 subjects (758 T2DM cases and 621 controls) from Hyderabad, Andhra Pradesh. The T2DM patients were recruited from J.P Endocrine Center, Hyderabad, based on their medical record and adhering to ADA 2010 criteria [46], which stipulates fasting plasma glucose ≥126 mg/dl and/or 2 hr plasma glucose ≥200 mg/dl or casual plasma glucose (random blood sugar) ≥200 mg/dl. We conducted a free diabetic camp in different organizations in Hyderabad and individuals above the age of 40 years were tested for random blood sugar (where fasting and 2 hr plasma glucose were not feasible ) and those with random blood sugar values <140 mg/dl were recruited as controls. We collected background data on age at onset of T2DM, duration of T2DM since diagnosis, family history, and anthropometric measurements like height, weight, waist and hip circumferences and other parameters such as blood pressure of the cases. The above four anthropometric measurements were taken for both the cases and controls in addition to the family history, lifestyle parameters and health status.

About 3–5 ml blood was collected from both T2DM cases and controls. We isolated DNA using the phenol-chloroform method [47], which was quantified with the help of NanoDrop. The three SNPs of the TCF7L2 viz., rs7903146, rs12255372 and rs11196205, were genotyped on Sequenom Massarray platform [48] at The Centre for Genomic Application (TCGA), Delhi, during the year 2011. The raw data files generated by Mass Array Sequenom were analysed for the intensity peaks of calibrant to ascertain the quality of the data. An overall call rate of >95% was maintained. For every 96 samples (a quadrant of Sequenom chip), 4 samples were duplicated and the call rates were checked for concordance. The calls in the negative control (no DNA) was also monitored in all the runs.

Statistical analyses were performed with the help of SPSS statistical software (version 18.0, IBM SPSS). Data for continuous variables were expressed as mean± SD, median and range. Chi-square test was used to compare the allele and the genotype frequencies between the cases and the controls. Using ‘SNP association’ package of R PROGRAM, logistic regression analysis was done under the assumption of different genetic models viz., co-dominant, dominant, over-dominant, recessive and log additive, to explore the most parsimonious one that fits our data in case of each of the three SNPs. The above logistic regression procedure was repeated after adding BMI (Body Mass Index) and WHR (Waist to Hip Ratio) as covariates. Post-hoc power of the study was calculated using G*Power software (version 3.1). The Hardy-Weinberg equilibrium was determined through goodness of fit χ2 test using Pypop software. Haploview and THESIAS softwares were used to estimate LD and generate haplotype frequencies.

### Ethics Statement

The study was approved by the Indian Statistical Institute Review Committee for Protection of Research Risks to Humans and written informed consent was obtained from all the participants.

## Results

The clinical profile of the subjects, represented by mean, median and range of different variables such as age of the subjects, age at onset of the disease, BMI, WHR, FPG, PPG etc is presented in Table S1. Over all, the sample, albeit not purposive, represents a relatively greater proportion of male subjects as compared to females, in the ratio of ∼3∶2. The case and control samples represent similar age range, mean and median values. However, the mean and median values of BMI, WHR and RBG of the cases were much higher than the controls and highly significantly different (p<0.01) from them.

Tables 1 and 2 show allele and genotype frequencies of the three SNPs of TCF7L2 considered for this study. Using PyPop software, the observed genotype counts were compared with those expected under Hardy–Weinberg proportions (HWPs) and the χ2 test suggest that all the three SNP loci conform to the Hardy-Weinberg equilibrium in both case and control samples. The risk allele frequency for each of the three SNPs – **T** of rs7903146(C/T), **C** of rs11196205 (G/C) and **T** of rs12255372 (G/T) - was found to be significantly higher in the T2DM cases than the normal controls, suggesting that they may confer risk for diabetes. The heterozygote and the risk homozygote frequencies were also higher in T2DM cases when compared to the controls.

**Table 1:** Allelic frequency distribution and allelic OR from the Logistic regression of T2DM on the risk allele frequency.

SNP	Allele	Cases(N = 758)	Controls(N = 621)	*χ2*	O.R (95% C.I)	p-value
**rs7903146**	C	0.67	0.79	46.9	**1.89 (1.57–2.26)**	**<0.001**
	**T**	**0.33**	**0.21**			
**rs11196205**	G	0.61	0.65	6.51	**1.23 (1.05–1.45)**	**0.011**
	**C**	**0.39**	**0.35**			
**rs12255372**	G	0.76	0.82	17.93	**1.51 (1.25–1.82)**	**<0.001**
	**T**	**0.24**	**0.18**			

**Table 2:** Genotypic frequency distribution of the three SNPs of TCF7L2 inT2DM cases and controls.

SNP	Genotype	Cases (N = 758)	Controls (N = 621)	χ2 p-value	*OR	95%C.I	p-value	adjusted for BMI and WHR		
								*OR	95%C.I	p-value
**rs7903146**	CC	0.45	0.63	45.96(<**0.001**)	**1.86**	**1.54–2.23**	**<0.001**	**1.85**	**1.53–2.24**	**<0.001**
	**CT**	**0.43**	**0.31**							
	**TT**	**0.11**	**0.05**							
**rs11196205**	GG	0.36	0.43	6.755(**0.034**)	**1.24**	**1.05–1.46**	**0.01**	**1.23**	**1.03–1.45**	**0.02**
	**GC**	**0.49**	**0.46**							
	**CC**	**0.15**	**0.12**							
**rs12255372**	GG	0.57	0.68	18.20(<**0.001**)	**1.50**	**1.24–1.82**	**<0.001**	**1.48**	**1.22–1.81**	**<0.001**
	**GT**	**0.37**	**0.28**							
	**TT**	**0.05**	**0.04**							

* Odds ratios from Logistic regression under additive model.

The logistic regression yielded significant odds ratio (Table 1) suggesting that the risk allele confers significant risk for developing T2DM with reference to each of the three SNPs: rs 7903146 (OR 1.88, p<0.001); rs11196205 (OR 1.23, p = 0.011) and rs12255372 (OR 1.50, p<0.001). The genotypic OR from Logistic regression under additive model also showed significant association (Table 2) of rs7903146 and rs12255372 (OR = 1.86, p = <0.001 and OR = 1.54, p = <0.001,respectively) with T2DM. Although the second SNP (rs11196205) also showed significant association (OR = 1.24, p = 0.001), it was not as strong as in case of the other two SNPs.

Logistic regression analysis of T2DM on the genotypes of each of the three SNPs was repeated by using BMI and WHR as covariates under additive model to explore if the observed association of the SNPs with T2DM is not due to the confounding nature of association of the covariates with the genotypes as well as the disease phenotype. The results of this analysis (Table 2) suggest that, after adjusting for covariates, there has been virtually no change in the status of association, especially in case of SNP genotypes pertaining to rs7903146 and rs11196205 implying that the genetic association observed with reference to TCF7L2 SNP genotypes is independent of the effect of covariates. However, after removing the covariate effects, the strength of association was diminished in case of the third SNP rs12255372. Given large sample size, our study shows high statistical power (1−β error probability) of 99–100%. However, the mean BMI did not significantly vary among genotypes, either in cases or control presented in Table S2 albeit, as expected, significant mean difference in BMI was evident (p<0.01) between cases and controls with reference to each genotype of the respective SNPs. We did not find significant association of age and gender as covariates with T2DM. In an effort to validate these results in the same population and to check internal consistency of our results we have split our sample into 30%, 50% and 70% random subsets of cases and controls and repeated the analysis (i.e. allele, genotype frequencies, Logistic regression of respective alleles and logistic regression of genotypes under additive model of all the three SNPs). The results showed similar association pattern with T2DM with reference to both alleles (Table S3) and genotypes (Table S4) in each of the three subsets as compared to the total sample, showing internal consistency.

**Figure 1:**
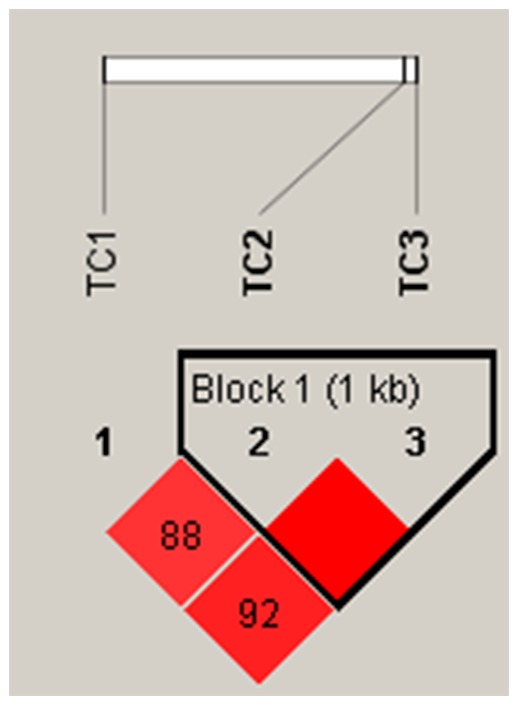
Linkage disequilibrium plot for TCF7L2 SNPs in the pooled sample of T2DM cases and controls. (*TC1 = rs7903146; TC2 = rs11196205, TC3 = rs12255372) D' values are mentioned in the LD blocks.

On the other hand, while rs11196205 and rs12255372 are in perfect LD with D′ = 1, r^2^ = 0.46 the other two combinations of rs7390146: rs11196205 and rs7390146: rs12255372 also show strong LD with D′ of 0.88 and 0.92 (Figure 1) and with r^2^ value of 0.53 and 0.58, respectively. The 121, 221 and 222 haplotypes, with risk alleles, show relatively greater difference in frequency between cases and controls as compared to the others (Tables 3&4). The logistic regression analysis, using the most prevalent wild haplotype (111 i.e. CGG) as the reference, revealed significant OR for 121, 221 and 222 haplotypes (Table 3); where as 121 is protective, 221 and 222 are risk conferring in nature. However, after Bonferroni correction for multiple testing, only the two risk conferring haplotypes (222 and 221) were retained as significantly associated with T2DM. It is indeed pertinent to note that the most strongly associated and risk conferring haplotype, 222, is a combination of only risk alleles of the three SNPs, the haplotype 221 represents risk alleles of two of the three SNPs (rs7390146 and rs11196205). A similar pattern of association was apparent when logistic regression was performed using BMI and WHR as covariates (Table 4), albeit with marginally increased strength in case of all risk alleles 222 and the protective 121 haplotypes, which retain significance even after Bonferroni correction.

**Table 3:** Haplotype frequencies in T2DM cases and controls and the results of haplotype based logistic regression analysis.

Haplotype	Haplotype code	Cases	Controls	#OR	(95% C.I)	p≤
**CGG**	111	0.58	0.65	–	–	–
**CCG**	**121**	**0.08**	**0.12**	**0.73**	**0.55–0.96**	**0.03**
**CCT**	122	0.01	0.01	0.62	0.29–1.34	0.23
**TGG**	211	0.02	0.01	1.98	1.00–3.91	0.05
**TCG**	**221**	**0.07**	**0.05**	**1.68**	**1.17–2.42**	**0.01** *
**TCT**	**222**	**0.24**	**0.16**	**1.62**	**1.32–1.99**	**0.001** *

# Haplotypic OR is in comparison to the reference haplotype 111, which is constituted by the wild type alleles of the three SNPs and shows in maximum frequency.

* Significant even after Bonferroni Correction.

**Table 4:** Haplotype frequency of T2DM Cases and Controls and the results of logistic regression of T2DM on the haplotypes, using BMI and WHR as covariates.

Haplotype	*Haplotype* Code	*Covariate*	*Cases*	*Controls*	# *OR*	*(95% C.I)*	*p value*
		***BMI***			***1.10***	***1.08–1.12***	***<0.01***
		***WHR***			***0.01***	***0.002–0.014***	***<0.01***
**CGG**	111		0.58	0.65	–	–	–
**CCG**	**121**		**0.07**	**0.12**	**0.68**	**0.50–0.92**	**0.01** *
**CCT**	122		0.01	0.01	0.62	0.29–1.32	0.21
**TGG**	211		0.02	0.01	2.09	0.99–4.41	0.05
**TCG**	**221**		**0.07**	**0.05**	**1.54**	**1.04–2.26**	**0.03**
**TCT**	**222**		**0.23**	**0.15**	**1.60**	**1.29–1.98**	**0.000021** *

# Haplotypic OR is in comparison to the reference haplotype 111, which is constituted by the wild type alleles of the three SNPs and shows in maximum frequency.

* Significant even after Bonferroni Correction.

The risk allele frequency of the three SNPs, rs7903146, rs11196205 and rs12255372 observed in our population and the range of allele frequency for other Indian and Non-Indian populations are presented in Table S5. Broadly speaking, it is within the range observed for Indian populations albeit at the lower end. Further, the range of risk allele frequency falls within the range for non-Indian populations. Although the very few studies hitherto conducted on vastly heterogenous population of India precludes scope for any meaningful meta-analysis of the data generated on the three SNPs, we have compiled population and region specific data on allele frequency and odds ratio to gauge the possible heterogeneity in the allele frequency and the nature and magnitude of its association with T2DM (Table 5). Unfortunately, while 6 populations were studied for rs7903146 (two from south [17], two from North [14], [15] and two independent populations from Pune [13], [14]), only one more population (besides ours from Hyderabad) from North [15] for rs11196205 and three more for rs12255372 were screened representing south [17], north [15] and Pune [13] from mid-west India. Nevertheless, the test of homogeneity of allele frequency across Indian populations so far studied suggest significant heterogeneity of allele frequency for each of the three SNPs in the pooled sample of cases and controls *(rs7903146: χ2 = 62.01, df = 5., p<0.0001; rs11196205: χ2 = 44.5 df = 1, p<0.0001; rs12255372: χ2 = 62.4 df = 3, p<0.0001).* The heterogeneity observed is, to a large extent, because of the differences in allele frequency between the ethnically, linguistically as well as geographically distinct northern and southern populations (Table 5). These results were qualitatively similar when cases and controls were separately analyzed. This would suggest the nature of genetic heterogeneity implicit in the complex Indian population underscoring the importance of exploring a number of geographically and ethnically heterogenous populations before one can project representative picture on the genetic susceptibility of Indian population as a whole.

**Table 5:** Comparative presentation of Risk allele frequency (RAF), OR and C.I of the three SNPs of TCF7L2 gene among Indian populations.

Reference	Indian Populations	Sample size		rs7903146(C/*T)			rs11196205(G/C)			rs12255372(G/T)		
		Cases	Controls	RAF	OR(p-value)	C.I	RAF	OR(p value)	C.I	RAF	OR(p value)	C.I
*Our study*	**Hyderabad**	758	621	0.33	1.89(<0.001)	1.57–2.26	0.39	1.23(0.01)	1.05–1.45	0.24	1.51(<0.001)	1.25–1.82
*Chandak et al (2007)* [*^13^*]	**Pune**	955	399	0.37	1.46(3×10^−5^)	1.22–1.75	–	–	–	0.30	1.50(4×10^−5^)	1.24–1.82
*Bodhini et al (2007)* [*^17^*]	**Chennai**	1031	1038	0.33	1.29(0.0001)	1.13–1.48	–	–	–	0.23	1.30(0.001)	1.11–1.51
*Sanghera et al (2008)* [*^15^*]	**Khatri Sikhs - North India (** ***Punjab, Haryana, Himachal Pradesh, Delhi, Jammu &Kashmir*** **)**	556	536	0.41	− ∧1.39(0.010)	− ∧1.08–1.79	0.52	− ∧1.44(0.01)	− ∧1.09–1.92	*0.37*	# *NS* ∧ *1.19*	*0.93–1.52*
*Chauhan et al (2010)* [*^14^*]	**Delhi**	1019	1006	0.40	1.67(1.7×10^−13^)	1.46–1.92	–	–	–	–	–	–
	**Pune**	1467	1672	0.37	2.10(1.7×10^−17^)	1.77–2.49	–	–	–	–	–	–
	**combined** **(Delhi+Pune)**	2486	2678	0.38	1.89(4×10^−34^)	1.71–2.09	–	–	–	–	–	–

* Risk allele highlighted in bold.

# Non-significant.

∧ Genotypic adjusted OR,C.I and p value under dominant model only for this study as allelic odds ratio was not available.

## Discussion

TCF7L2 gene which spans about 215.9 Kb with 17 exons is a high mobility group box containing transcription factor involved in the Wnt signaling pathway, playing a key role in cell development and regulatory mechanisms. WNT activity is important for lipid and glucose metabolism, pancreatic beta cell proliferation and function and for production of incretin hormone GLP-1 [49]. WNT signaling is also critical for glucagon like peptide-1 (GLP-1) secretion by the intestinal endocrine L-cells [50]. Thus, alteration in this pathway could lead to reduced secretion of GLP-1 which, in turn, could have effect on insulin secretion. The GLP-1, in concert with insulin, plays a critical role in blood glucose homeostasis. It has been postulated that TCF7L2 gene variants may confer susceptibility to T2DM indirectly by altering GLP-1 levels [51]. It was predicted that in the carriers of TCF7L2 risk alleles, the Wnt signaling is potentially increased and thereby the pre-adipocytes are not differentiated into the mature adipocytes and probably influence the growth and development of adipose tissue and alter BMI [51], [52]. Thus TCF7L2 might play some role in the adipogenesis resulting in the deposition of triglycerides in peripheral tissues leading to insulin resistance [20].

Consistent with the functional relevance of this gene, the results of our study are confirmatory to the earlier findings among the other Indian [13]–[17] as well as non-Indian populations [3], [19]–[31] in that they show strong association of TCF7L2 variants with increased risk of T2DM. In general, the association of TCF7L2 with T2DM is reiterated in our population not only with reference to risk alleles of the SNPs studied but also in case of genotypes of each SNP (under additive model) and haplotypes derived from the three SNPs which are in strong LD. Thus our results reiterate TCF7L2 as the most promising T2DM susceptible gene that has been most universally replicated. While the risk allele of each of the three TCF7L2 SNPs was highly significantly associated with T2DM, the greatest risk of developing the disease was conferred by rs7903146, which is consistent with the findings from other Indian studies [13]–[17] as well as migrant Indians living in other countries [53]. Further, the risk conferred by homozygotes is much higher than the heterozygote carriers and it is marked in case of rs7903146. Thus our study not only shows consistency with the earlier findings in case of the association of risk alleles but also conforms to the pattern of multiplicative model of inheritance hypothesized by Grant et al (2006) in which the risk conferred by homozygotes was higher than that of the heterozygotes for all the SNPs. In somewhat a concurrent pattern, the three SNPs of TCF7L2, which are located on the chromosome 10q25.3, show strong LD and the logistic regression analysis based on haplotype frequency suggest that haplotype 222 which carries all the three risk alleles of the SNPs (rs7903146, rs11196205 and rs12255372) is most significantly associated with T2DM risk as compared to the others with either one or two of the risk alleles.

The confounding nature of the influence of environmental factors in the manifestation of T2DM is confirmed by the significant association of BMI and WHR as covariates in the Logistic Regression Analysis of T2DM on genetic variants of TCF7L2, albeit a highly significant association was evident for rs7903146 and rs11196205 even after adjusting for the effect of covariates. However, the other SNP (rs12255372) shows somewhat decreased risk, after adjusting for covariates, probably suggesting the confounding effect of environmental factors to be relatively greater with reference to this gene. The mean difference of BMI between cases and controls was highly significant for each genotype of respective SNPs, indirectly suggesting greater body fat of T2DM patients, in general, albeit inter genotype differences in mean BMI was not evident for any of the three SNPs. Despite TCF7L2 being the most promising T2DM susceptible gene, the precise mechanism of action of TCF7L2 SNPs in the etiology of T2DM is still unclear as all the SNPs of TCF7L2 identified so far are found in the intronic regions. It is necessary to understand how these intronic SNPs affect the expression of TCF7L2. Interestingly, none of the variants found in the exonic regions were associated with the T2DM. Earlier studies focusing on the function of TCF7L2 could trace expression to the gut rather than to the pancreatic islets. Although Lyssenko et al (2007) showed an increased gene expression of TCF7L2 in risk allele carriers in the pancreatic islets, certain controversies still exist with the increased or decreased expression of this gene. Therefore, research focusing on the functional relevance of this gene is of importance and more extensive studies are needed to study whether the variants of TCF7L2 are involved in the alternative splicing, gene expression or protein structure.

Given that the population of this region was not genetically explored hitherto for any of the complex genetic disorders, T2DM in particular, it is necessary to assess the role of different candidate genes in the etiology of T2DM in Andhra Pradesh and other regions of India with high prevalence of T2DM, to gauge the heterogeneity in the susceptible genetic profile of the Indian population, in general, which is characterized by enormous geographic, ethnic, cultural and genetic heterogeneity. We consider that our study contributes to this end significantly, even if it appears to only reiterate the oft observed strong association of the TCF7L2 SNPs with the risk of developing T2DM in the diverse populations of the world, reiterating universality of its association across ethnic groups. Further, our study could have also helped in understanding the effect of ethnicity, if existent, on the T2DM susceptible genes.

## Supporting Information

Table S1 **Clinical profile of the T2DM cases and controls.** Footnote: *Numbers in the parentheses are the SDs. ^∧^NA = Not Applicable and ^#^NI = No information
(DOC)

Table S2 **Mean BMI according to genotypes of TCF7L2 SNPs (rs7903146, rs11196205, rs12255372) and the values of t test for mean difference between cases and controls.** Footnote: P values *<0.01, **0.001 and ***<0.001. F values are not significant for all the SNPs (not mentioned in the table)
(DOC)

Table S3 **Subset analysis (30%,50%,70%) of T2DM cases and controls for the TCF7L2 SNPs (rs7903146, rs11196205, rs12255372) showing allelic frequency distribution and OR using logistic regression.**
(DOCX)

Table S4 **Subset analysis (30%,50%,70%) of T2DM cases and controls for the three SNP’s (rs7903146, rs11196205, rs12255372) showing genotype frequencies and genotypic OR using logistic regression under additive model.**
(DOCX)

Table S5 **Risk Allele frequency of SNPs rs7903146, rs11196205 and rs12255372 of TCF7L2 in the population of A.P and among other Indian and Non-Indian populations.** Footnote: Risk Allele frequency is presented as range for the other Indian and Non-Indian populations. The RAF which is unusually low in the ^##^Japanese and ^##^Chinese samples is not included in the range. *Genomic position of the respective SNPs on chromosome 10 and allelic nomenclature is according to NCBI dbSNP Build 37. **Risk Alleles marked as bold. ***Odds ratio range specified for only Indian populations
(DOC)
