# Supplementary material for: Association of TCF7L2 Gene Polymorphisms with T2DM in the Population of Hyderabad, India
Source: PLoS One. 2013 Apr 5;8(4):e60212. doi: 10.1371/journal.pone.0060212 (PMC3618330; doi:10.1371/journal.pone.0060212)
Supplement: Table S1 — Clinical profile of the T2DM cases and controls. Footnote: *Numbers in the parentheses are the SDs. ∧NA = Not Applicable and #NI = No information (DOC) [file pone.0060212.s001.doc]

**Table S1**

|  | **Mean*** | | **Median** | | **Range** | |
| --- | --- | --- | --- | --- | --- | --- |
|  | ***CASES(N=758)M/F(443/315)*** | ***CONTROLS(N=621)M/F(395/226)*** | ***CASES(N=758)*** | ***CONTROLS(N=621)*** | ***CASES(N=758)*** | ***CONTROLS(N=621)*** |
| ***Age*** | *52.5(9.08)* | *52.20(7.55)* | *52.00* | *50.00* | *31-80* | *40-85* |
| ***Age at onset (yrs)*** | *44.78(8.65)* | *^NA* | *45.00* | *NA* | *25-71* | *NA* |
| ***Duration of T2DM since diagnosis(yrs)*** | *9.18(7.69)* | *NA* | *7.00* | *NA* | *1-38* | *NA* |
| ***BMI*** | *27.07(4.63)* | *24.72(4.62)* | *26.5* | *24.80* | *13.0-44.1* | *12.8-29.0* |
| ***WHR*** | *1.69(0.43)* | *0.94(0.03)* | *1.67* | *0.95* | *0.55-3.51* | *0.72-1.09* |
| ***FPG(mg/dl)*** | *115.50(32.45)* | *NA* | *108.00* | *NA* | *47-323* | *NA* |
| ***PPG(mg/dl)*** | *195.71(52.83)* | *NA* | *191.00* | *NA* | *82-409* | *NA* |
| ***RBG(mg/dl)*** | *199.27(81.60)* | *110.65(15.93)* | *185.50* | *108.00* | *73-520* | *70-139* |
| ***SBP(mmHg)*** | *128.11(10.73)* | *^#^NI* | *130.00* | *NI* | *94-180* | *NI* |
| ***DBP(mmHg)*** | *80.02(8.57)* | *NI* | *80.00* | *NI* | *20-215* | *NI* |
