# Supplementary material for: Association of TCF7L2 Gene Polymorphisms with T2DM in the Population of Hyderabad, India
Source: PLoS One. 2013 Apr 5;8(4):e60212. doi: 10.1371/journal.pone.0060212 (PMC3618330; doi:10.1371/journal.pone.0060212)
Supplement: Table S2 — Mean BMI according to genotypes of TCF7L2 SNPs (rs7903146, rs11196205, rs12255372) and the values of t test for mean difference between cases and controls. Footnote: P values *<0.01, **0.001 and ***<0.001. F values are not significant for all the SNPs (not mentioned in the table) (DOC) [file pone.0060212.s002.doc]

**Table S2**

| **SNP** | **CASES** | | | | | **CONTROLS** | | | |  |
| --- | --- | --- | --- | --- | --- | --- | --- | --- | --- | --- |
|  | **GENOTYPES** | **N** | **MEAN** | **S.E** | **95% C.I** | **N** | **MEAN** | **S.E** | **95%C.I** | **t TEST** |
| **rs7903146** | **CC** | 270 | 26.81 | 0.27 | 26.29-27.33 | 319 | 24.73 | 0.26 | 24.22-25.24 | ***5.55 |
|  | **CT** | 279 | 27.16 | 0.26 | 26.65-27.67 | 167 | 24.31 | 0.36 | 23.60-25.01 | ***6.42 |
|  | **TT** | 67 | 26.98 | 0.53 | 25.93-28.02 | 26 | 23.68 | 0.91 | 21.90-25.47 | *3.13 |
|  | **TOTAL** | 616 | 26.99 | 0.22 | 26.56-27.41 | 512 | 24.24 | 0.34 | 23.58-24.90 | ***6.79 |
| **rs11196205** | **GG** | 229 | 26.80 | 0.29 | 26.23-27.36 | 236 | 24.80 | 0.30 | 24.21-25.39 | ***4.79 |
|  | **GC** | 298 | 27.00 | 0.25 | 26.50-27.50 | 218 | 24.25 | 0.31 | 23.64-24.87 | ***6.91 |
|  | **CC** | 89 | 27.41 | 0.46 | 26.50-28.31 | 58 | 24.52 | 0.61 | 23.33-25.72 | ***3.78 |
|  | **TOTAL** | 616 | 27.07 | 0.20 | 26.68-27.46 | 512 | 24.53 | 0.25 | 24.04-25.02 | ***7.93 |
| **rs12255372** | **GG** | 352 | 26.89 | 0.23 | 26.44-27.35 | 362 | 24.61 | 0.24 | 24.13-25.08 | ***6.86 |
|  | **GT** | 228 | 26.91 | 0.29 | 26.34-27.47 | 130 | 24.42 | 0.41 | 23.62-25.22 | **4.96 |
|  | **TT** | 36 | 28.41 | 0.73 | 26.98-29.83 | 20 | 23.96 | 1.04 | 21.92-26.00 | ***3.50 |
|  | **TOTAL** | 616 | 27.40 | 0.27 | 26.87-27.93 | 512 | 24.33 | 0.38 | 23.58-25.07 | ***6.59 |
