# Supplementary material for: Association of TCF7L2 Gene Polymorphisms with T2DM in the Population of Hyderabad, India
Source: PLoS One. 2013 Apr 5;8(4):e60212. doi: 10.1371/journal.pone.0060212 (PMC3618330; doi:10.1371/journal.pone.0060212)
Supplement: Table S3 — Subset analysis (30%,50%,70%) of T2DM cases and controls for the TCF7L2 SNPs (rs7903146, rs11196205, rs12255372) showing allelic frequency distribution and OR using logistic regression. (DOCX) [file pone.0060212.s003.docx]

**Table S3**

| **SNP** | **%Sample (Cases, Controls)** | **Allele** | **Cases** | **Controls** | **p value** | **O.R (95%C.I)** |
| --- | --- | --- | --- | --- | --- | --- |
| **rs7903146** | 30 | C | 0.65 | 0.78 | <0.001 | 1.85 (1.32-2.58) |
|  |  | T | 0.35 | 0.22 |  |  |
|  | 50 | C | 0.65 | 0.78 | <0.001 | 1.92 (1.47-2.50) |
|  |  | T | 0.35 | 0.22 |  |  |
|  | 70 | C | 0.65 | 0.79 | <0.001 | 2.03 (1.58-2.63) |
|  |  | T | 0.35 | 0.21 |  |  |
| **rs11196205** | 30 | G | 0.57 | 0.67 | <0.001 | 1.54 (1.13-2.10) |
|  |  | C | 0.43 | 0.33 |  |  |
|  | 50 | G | 0.61 | 0.69 | 0.004 | 1.42 (1.12-1.82) |
|  |  | C | 0.39 | 0.31 |  |  |
|  | 70 | G | 0.59 | 0.68 | 0.002 | 1.44 (1.15-1.82) |
|  |  | C | 0.41 | 0.32 |  |  |
| **rs12255372** | 30 | G | 0.74 | 0.84 | 0.001 | 1.93 (1.32-2.81) |
|  |  | T | 0.26 | 0.16 |  |  |
|  | 50 | G | 0.74 | 0.84 | <0.001 | 1.83 (1.37-2.46) |
|  |  | T | 0.26 | 0.16 |  |  |
|  | 70 | G | 0.75 | 0.84 | <0.001 | 1.74 (1.31-2.30) |
|  |  | T | 0.25 | 0.16 |  |  |
