# Supplementary material for: Association of TCF7L2 Gene Polymorphisms with T2DM in the Population of Hyderabad, India
Source: PLoS One. 2013 Apr 5;8(4):e60212. doi: 10.1371/journal.pone.0060212 (PMC3618330; doi:10.1371/journal.pone.0060212)
Supplement: Table S4 — Subset analysis (30%,50%,70%) of T2DM cases and controls for the three SNP’s (rs7903146, rs11196205, rs12255372) showing genotype frequencies and genotypic OR using logistic regression under additive model. (DOCX) [file pone.0060212.s004.docx]

| **SNP** | **%Sample (cases, controls)** | **Genotype** | **Cases** | **Controls** | **p value** | **O.R** | **C.I** | **p value** |
| --- | --- | --- | --- | --- | --- | --- | --- | --- |
| **rs7903146** | 30 | CC | 0.41 | 0.61 | 0.001 | 1.90 | 1.34-2.69 | 0.0002271 |
|  |  | CT | 0.49 | 0.34 |  |  |  |  |
|  |  | TT | 0.1 | 0.06 |  |  |  |  |
|  | 50 | CC | 0.43 | 0.63 | <0.001 | 1.87 | 1.44-2.44 | 1.544e-06 |
|  |  | CT | 0.45 | 0.31 |  |  |  |  |
|  |  | TT | 0.12 | 0.06 |  |  |  |  |
|  | 70 | CC | 0.43 | 0.63 | <0.001 | 1.98 | 1.54-2.56 | 4.052e-08 |
|  |  | CT | 0.44 | 0.31 |  |  |  |  |
|  |  | TT | 0.13 | 0.05 |  |  |  |  |
| **rs11196205** | 30 | GG | 0.33 | 0.47 | 0.021 | 1.52 | 1.12-2.06 | 0.006757 |
|  |  | GC | 0.48 | 0.40 |  |  |  |  |
|  |  | CC | 0.18 | 0.12 |  |  |  |  |
|  | 50 | GG | 0.37 | 0.48 | 0.017 | 1.43 | 1.12-1.82 | 0.004173 |
|  |  | GC | 0.48 | 0.42 |  |  |  |  |
|  |  | CC | 0.15 | 0.10 |  |  |  |  |
|  | 70 | GG | 0.35 | 0.47 | 0.005 | 1.44 | 1.14-1.81 | 0.001648 |
|  |  | GC | 0.49 | 0.42 |  |  |  |  |
|  |  | CC | 0.17 | 0.12 |  |  |  |  |
| **rs12255372** | 30 | GG | 0.55 | 0.73 | 0.002 | 1.85 | 1.28-2.68 | 0.0007805 |
|  |  | GT | 0.38 | 0.23 |  |  |  |  |
|  |  | TT | 0.07 | 0.04 |  |  |  |  |
|  | 50 | GG | 0.55 | 0.73 | 0.017 | 1.78 | 1.33-2.38 | 5.472e-05 |
|  |  | GT | 0.39 | 0.23 |  |  |  |  |
|  |  | TT | 0.07 | 0.05 |  |  |  |  |
|  | 70 | GG | 0.56 | 0.71 | <0.001 | 1.72 | 1.30-2.27 | 8.744e-05 |
|  |  | GT | 0.38 | 0.25 |  |  |  |  |
|  |  | TT | 0.06 | 0.04 |  |  |  |  |

**TableS4**
