# Supplementary material for: Association of TCF7L2 Gene Polymorphisms with T2DM in the Population of Hyderabad, India
Source: PLoS One. 2013 Apr 5;8(4):e60212. doi: 10.1371/journal.pone.0060212 (PMC3618330; doi:10.1371/journal.pone.0060212)
Supplement: Table S5 — Risk Allele frequency of SNPs rs7903146, rs11196205 and rs12255372 of TCF7L2 in the population of A.P and among other Indian and Non-Indian populations. Footnote: Risk Allele frequency is presented as range for the other Indian and Non-Indian populations. The RAF which is unusually low in the ##Japanese and ##Chinese samples is not included in the range. *Genomic position of the respective SNPs on chromosome 10 and allelic nomenclature is according to NCBI dbSNP Build 37. **Risk Alleles marked as bold. ***Odds ratio range specified for only Indian populations (DOC) [file pone.0060212.s005.doc]

**Table** S5

| ***SNP*** | ****Position on Chromosome 10*** | ***Location*** | *****Major/Minor*** | ***RAF in T2DM Cases*** | | |
| --- | --- | --- | --- | --- | --- | --- |
|  |  |  |  | ***Our study*** | ***Other Indian Studies*** | ***Non-Indian studies*** |
| ***rs7903146*** | *114758349* | *Intron3* | *C/****T*** | ***0.33(***1.89)*** | ***0.33-0.41****(*******1.29-1.89)****^[17,13,14,15]^* | ***0.22-0.49*** *^[19,29,21,20,26,30,31,3,37,36,32]^***^##^***0.05(Japanese)^[27]^* |
| ***rs11196205*** | *114797037* | *Intron4* | *G/****C*** | ***0.39(1.23)*** | ***0.52***^[15]^ | ***0.41-0.54****^[20,19,18,26,3]##^0.03(Chinese)^[24]^* |
| ***rs12255372*** | *114808902* | *Intron4* | *G/****T*** | ***0.24(1.51)*** | ***0.23-0.37(1.30-1.50)****^[17,13,15]^* | ***0.20-0.42****^[19,34,3,31,20]##^0.04(Japanese)^[26]^* |
